# BET Inhibitors Synergize with Carfilzomib to Induce Cell Death in Cancer Cells via Impairing Nrf1 Transcriptional Activity and Exacerbating the Unfolded Protein Response

**DOI:** 10.3390/biom10040501

**Published:** 2020-03-26

**Authors:** Janakiram R. Vangala, Ajay Potluri, Senthil K. Radhakrishnan

**Affiliations:** Department of Pathology and Massey Cancer Center, Virginia Commonwealth University, Richmond, VA 23298, USA; janakiram.vangala@vcuhealth.org (J.R.V.); potlurias@mymail.vcu.edu (A.P.)

**Keywords:** BET inhibitors, Nrf1, proteasome genes, transcription, proteasome inhibitor, cancer, unfolded protein response

## Abstract

Currently, proteasome inhibitors bortezomib, carfilzomib, and ixazomib are successfully used in clinics to treat multiple myeloma. However, these agents show limited efficacy against solid tumors. Identification of drugs that can potentiate the action of proteasome inhibitors could help expand the use of this therapeutic modality to solid tumors. Here, we found that bromodomain extra-terminal (BET) family protein inhibitors such as JQ1, I-BET762, and I-BET151 synergize with carfilzomib in multiple solid tumor cell lines. Mechanistically, BET inhibitors attenuated the ability of the transcription factor Nrf1 to induce proteasome genes in response to proteasome inhibition, thus, impeding the bounce-back response of proteasome activity, a critical pathway by which cells cope with proteotoxic stress. Moreover, we found that treatment with BET inhibitors or depletion of Nrf1 exacerbated the unfolded protein response (UPR), signaling that was initiated by proteasome inhibition. Taken together, our work provides a mechanistic explanation behind the synergy between proteasome and BET inhibitors in cancer cell lines and could prompt future preclinical and clinical studies aimed at further investigating this combination.

## 1. Introduction

The ubiquitin-proteasome system (UPS) is a major quality control pathway in eukaryotic cells and is responsible for selective and timely removal of proteins that are destined for destruction [1]. Selectivity of the UPS is achieved via tagging the substrates with ubiquitin chains which enables its recognition and processive degradation by the 26S proteasome, a multi-catalytic protease complex [2]. The 26S proteasome is composed of the 20S core particle and the 19S regulatory particle. The actual degradation of protein substrates occurs in the 20S core and is facilitated by its chymotrypsin-like, trypsin-like, and caspase-like activities conferred, respectively, by β5, β2, and β1 protein subunits of the proteasome. The 19S regulatory particle caps at one or both ends of the 20S proteasome and enables the recognition and transfer of ubiquitinated target proteins to the 20S catalytic core for degradation [3].

Rapidly proliferating cells such as cancer cells have an increased reliance on proteasome activity [4]. Hence, proteasome inhibitors have been found to be effective anticancer therapeutic agents in some settings [5]. To date, three proteasome inhibitors, bortezomib, carfilzomib, and ixazomib, have been approved by the FDA for clinical use against multiple myeloma (MM) and mantle cell lymphoma (MCL). These proteasome inhibitors mainly target the β5 chymotrypsin-like activity of the proteasome. However, the events downstream of β5 inhibition that lead to cancer cell death are not completely understood.

In the case of MM cells which rely heavily on the anti-apoptotic transcription factor NF-κB for their survival, proteasome inhibitors induce accumulation of IκB, an inhibitor of NF-κB. This inactivates the NF-κB pathway and has been proposed to be one of the reasons for MM’s susceptibility to proteasome inhibitors [6]. In addition, given that MM cells are professional secretory cells that specialize in producing high amounts of immunoglobulins, they are reliant on a stress signaling pathway called the unfolded protein response (UPR) and ER-associated degradation (ERAD) to handle misfolded immunoglobulin chains. Although UPR can be cytoprotective, hyperactivation of this pathway, as can be seen in MM cells treated with proteasome inhibitors, can lead to cell death, thus, offering another explanation for proteasome inhibitor action in MM [6]. In the case of MCL, proteasome inhibitors have been proposed to work via induction of oxidative stress and also through upregulation of NOXA, a pro-apoptotic Bcl-2 family member [7].

In addition to triggering apoptosis, proteasome inhibitors also invoke certain cytoprotective pathways such as the heath-shock response [8], autophagy [9,10,11], and transcription factor Nrf1-mediated proteasome ”bounce-back” response [12]. We and others have characterized the proteasome bounce-back response, wherein inhibition of the proteasome results in the activation of Nrf1 which directs transcriptional upregulation of proteasome genes, thus, mitigating the cellular proteotoxic stress [13,14,15,16]. Overall, the interplay between the cytotoxic and cytoprotective effects elicited by the proteasome inhibitors determines the cell fate. 

Despite the reasonable success of proteasome inhibitors in treating MM and MCL patients, it is not clear why these agents are largely ineffective against solid tumors. Initially, this phenomenon was blamed on poor tumor penetration of the drug, based on studies from bortezomib [17]. However, ixazomib, which exhibits better tumor penetration, has not fared well in clinical trials with solid tumors [18]. A more recent view is that proteasome inhibitors need to be used in combination with other drugs to increase their efficacy in solid tumors [19]. Consistent with this idea, a number of ongoing clinical trials in solid tumors that evaluate proteasome inhibitors also include a second drug in combination. Several preclinical studies also support this notion. For instance, a recent report demonstrated that inactivation of the β2 site of the proteasome substantially increased the efficacy of bortezomib and carfilzomib in triple-negative breast cancer cell lines, as well as xenograft tumor models in mice [20]. Other studies have also shown that the combination of the chemotherapeutic agent doxorubicin with either carfilzomib or ixazomib enhanced cell killing in breast cancer cells [21,22]. Similarly, in head and neck, as well as pancreatic cancer cells, HDAC inhibitors potentiated bortezomib-induced cell death [23,24].

In this study, to expand the utility of proteasome inhibitors in treating solid tumors, we undertook a search for drugs that could synergize with carfilzomib and found that BET inhibitors fulfil this criterion in multiple solid tumor cell lines. We further explored the mechanism behind this phenomenon and found that BET inhibitors attenuated the Nrf1-mediated proteasome bounce-back response and also exacerbated proteasome inhibitor-induced UPR, thus, explaining the synergy between these agents.

## 2. Materials and Methods 

### 2.1. Cell Lines and Culture Conditions

All wild-type cell lines used in this study, i.e., A549, HCT116, MDA-MB-231, DU145, MIAPaCa2, T98G, and NIH-3T3 were from the American Type Culture Collection (ATCC). The generation of Nrf1 knockout cell line (NIH-3T3-Nrf1^KO^) was described previously in [16].The MDA-MB-231 with Nrf1 depletion (shNrf1) and a corresponding control cell line with pRS-puro vector were also reported previously in [12]. Dulbecco’s modified Eagle’s medium (DMEM) supplemented with 10% fetal bovine serum (Atlanta Biologicals), penicillin and streptomycin (Invitrogen) was used for all cell lines which were maintained at 37 °C in a humidified incubator with 5% CO_2_ [25,26,27,28]. 

### 2.2. MTT Assays for Measuring Cell Viability

The tetrazolium-based MTT assay was carried out as described in [29,30,31] with minor modifications. Briefly, 2 × 10^4^ cells/well were seeded into 96-well plates. Cells were treated with different concentrations of CFZ (0.5, 2, 8, and 32 nM) or BET inhibitors (I-BET762 and I-BET151) (100, 400, 1600, and 6400 nM) for 72 h in triplicates. For the assay, 10 µL of MTT (5 mg/mL) was added to each well and incubated for 1 h, followed by two washes with PBS. To dissolve the formazan crystals, 100 µl of DMSO was added to each well and absorbance was measured at 560 nm using a GloMax Explorer (Promega, Madison, WI, USA) microplate reader. Values were normalized to DMSO treated control cells. 

### 2.3. Determination of CI

Cells were treated with 4 concentrations of CFZ each in combination with 4 combinations of BET inhibitors in four-fold increments, as indicated above. This yielded a total of 16 different combinations and 10 different ratios. Each drug was also treated alone at these concentrations. All the treatments were done in triplicates. Fa (fraction affected, inhibition of cell proliferation) was calculated for all individual and cotreatments and used to determine CI (combination index) using CompuSyn software (ComboSyn Inc., Paramus, NJ, USA) which employs the Chou–Talalay method [32].

### 2.4. Quantitative Reverse Transcription PCR

Total RNA was isolated using TRIzol reagent (Invitrogen, Waltham, MA, USA) and quantitative reverse transcription PCR was carried out, as described previously in [15,16]. Briefly, 1000 ng of total RNA was used for making cDNA using iScript cDNA synthesis kit (Bio-Rad, Hercules, CA, USA). Then, quantitative PCR was carried out with iTaq universal SYBR green supermix (Bio-Rad) using a C1000 Touch Thermal cycler (Bio-Rad). CFX manager 3.1 (Bio-Rad) was used for the analysis of data. 18S rRNA levels were used for normalization. Primers used for the assays are listed in Table S1.

### 2.5. Proteasome Activity Recovery Assay

Measurement of proteasome activity was performed, as described previously in [15]. Briefly, about 90% of chymotrypsin-like proteasome activity of cells was inhibited by treatment with 20 nM carfilzomib for 1 h, followed by three times washing with PBS to remove residual CFZ. Then, cells were allowed to recover in fresh medium with or without BET inhibitors. At regular time points, the cells were frozen in TE buffer (20 mM Tris pH 8.0 and 5 mM EDTA). To measure chymotrypsin-like proteasome activity, the cell lysates obtained by freeze-thaw lysis were incubated with succinyl-Leu-Leu-Val-Tyr-amino-4-methylcoumarin (Suc-LLVY-AMC), and the resulting fluorescence was measured at 360/460 nm excitation/emission. The fluorescence values were then normalized by cell number which was quantitated using a Cell-Titer Glo kit (Promega, Madison, WI, USA) which measures the ATP levels in the cell.

### 2.6. Luciferase Assays

The 8xARE-Luc construct was generated by cloning 8 copies of the antioxidant response element [15] into the pGL3-promoter vector (Promega) that contains the firefly luciferase gene. Cells were transfected with the 8xARE-Luc construct along with a renilla luciferase construct (pRL-TK, Promega). Forty-eight hours after transfection, cells were subjected to overnight drug treatments and luciferase assays were performed using the Dual-Glo luciferase assay system (E2940, Promega). The firefly luciferase activity was, then, normalized to the renilla luciferase activity.

### 2.7. Immunoblot Analysis

Following treatments, cells were washed twice with PBS and lysed in 1× Laemmli sample buffer and subjected to immunoblot analysis as described previously [33]. Briefly, proteins were separated on SDS-PAGE, followed by transfer on to PVDF membrane. The membrane was washed twice with Tris-buffered saline with Tween before blocking with 5% non-fat dry milk (BioRad) for 1 h at room temperature, followed by overnight incubation with appropriate primary antibodies at 4 °C. The membrane was, then, washed three times and incubated with secondary antibody for 1 h at room temperature. Following a final set of three washes, the membrane was used for detection of the chemiluminescent signal using Supersignal West Dura substrate (Thermo Fisher Pierce, Waltham, MA, USA). The primary antibodies used were specific for Nrf1 (1:5000), BiP (1:2500), ATF6 (1:2500), ubiquitin (1:3000), PERK (1:3000), pEIF2α (1:2500), GADD34 (1:2500), CHOP (1:2000), XBP1s (1:2000), cleaved caspase-3 (1:3000) (all from Cell Signaling, Danvers, MA, USA), β-Actin (1:10,000, Sigma-Aldrich (Burlington, MA, USA). The secondary antibodies used were rabbit IgG HRP, and mouse IgG HRP (1:10,000; both from Bio-Rad).

### 2.8. RNA Sequencing and Analysis

Cells treated with CFZ or DMSO (vehicle control) were used for RNA extraction with a RNeasy mini kit (Qiagen, Germantown, MD, USA). A KAPA stranded mRNA-seq kit (Illumina, San Diego, CA, USA) was used for library preparation and run on 2 × 125 bp v4 high output lanes on Illumina HiSeq 2500 instrument. The library preparation and sequencing steps were carried out at the DNA sequencing core of the Brigham Young University. Then, differential expression of the genes was determined with the Illumina Base Space RNA Express app (Illumina, San Diego, CA, USA) that integrates STAR aligner [34] and DESeq2 [35,36] software packages.

### 2.9. Statistical Analysis

Data are presented as mean ± SD of at least three experiments, performed as a minimum in triplicate. Two-way Anova analysis was used to calculate *p* values for pairwise comparisons and *p* < 0.05 was considered to be significant. 

## 3. Results

### 3.1. Identification of BET Inhibitors as Synergizers of Proteasome Inhibitor-Induced Cancer Cell Death

**Figure 1:**
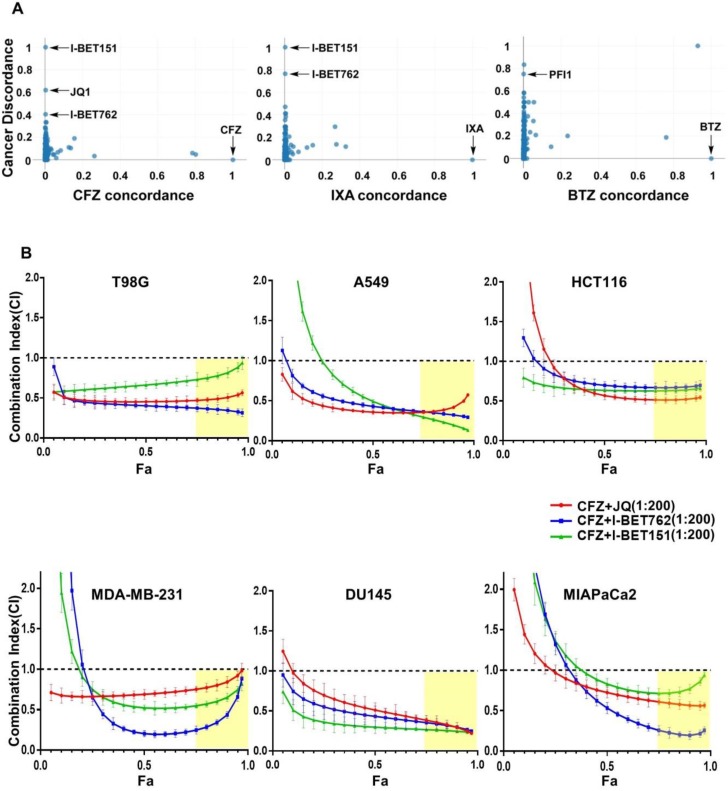
Synergistic interaction between proteasome and BET inhibitors in various cancer cells. (**A**) SynergySeq online platform was used to identify potential drugs that can synergize with proteasome inhibitors in cancer. “cancer discordance”, a measure of the ability of a drug to reverse cancer gene expression signature to a normal state, is shown on the y-axis. The level of similarity of a drug to the reference proteasome inhibitor drugs carfilzomib (CFZ), ixazomib-citrate (IXA), and bortezomib (BTZ) is shown as “concordance” values on the x-axis; (**B**) T98G, A549, HCT116, MDA-MB-231, DU145, and MIAPaCa2 cells were treated with different doses of CFZ (0.5, 2, 8, and 32 nM), along with one of the BET inhibitors (I-BET762, I-BET151, and JQ1) in different doses (0.1, 0.4, 1.6, and 6.4 μM) as indicated for 72 h. In these combination treatments, the ratio of CFZ to BET inhibitors was maintained at 1:200. The combination index (CI) and fraction affected (Fa) values were determined using CompuSyn software from cell viability data and are shown in these plots. The results are shown as mean ± SD, n = 3. CI < 1.0 indicates synergism, CI = 1.0 indicates additive effect, and CI > 1.0 indicates antagonism. The regions highlighted in yellow are synergistic (CI < 1.0) at optimal Fa > 0.75.

We used a recently described online platform, SynergySeq [37], to search for drugs that can synergistically interact with proteasome inhibitors. SynergySeq integrates glioblastoma gene expression data from The Cancer Genome Atlas (TCGA) [38] together with multi-cell line drug response data from the Library of Integrated Network-Based Cellular Signatures (LINCS) [39]. Given an input drug, this resource enables the identification of other drugs that can synergistically reverse the cancer gene expression to a more “normal state” in glioblastoma [37]. Using carfilzomib (CFZ), ixazomib-citrate (IXA), and bortezomib (BTZ) as input drugs in SynergySeq, we observed that various BET inhibitors such as I-BET151, JQ1, I-BET762, and PFI1 emerged as potential synergistic interactors with proteasome inhibitors (Figure 1A). 

To experimentally verify this prediction, first, we treated a glioblastoma cell line T98G with different concentrations of CFZ in combination with each of the BET inhibitors JQ1, I-BET762, and I-BET151. Then, we analyzed the resultant cell viability data using the established Chou-Talalay method, wherein a combination index (CI) value less than 1.0 is regarded synergistic [32]. Given that the fraction affected (Fa) is a measure of cell viability, we considered Fa values greater than 0.75 to be optimal. Using these criteria, we found several optimal CFZ + BET inhibitor combinations that were highly synergistic in the T98G cell line (Figure 1B; first panel). In order to test if this effect is true for cell lines derived from other tumor types, we employed A549 (lung), HCT116 (colon), MDA-MB-231 (breast), DU145 (prostate), and MIAPaCa2 (pancreatic) cell lines in a similar experiment. Indeed, we could find several optimal CFZ + BET inhibitor synergistic combinations in all of these cell lines (Figure 1B; panels 2–6), implying that this could be a general phenomenon independent of cancer type.

### 3.2. BET Inhibitors Attenuate CFZ-Mediated Nrf1-Dependent Proteasome Bounce-Back Response

**Figure 2:**
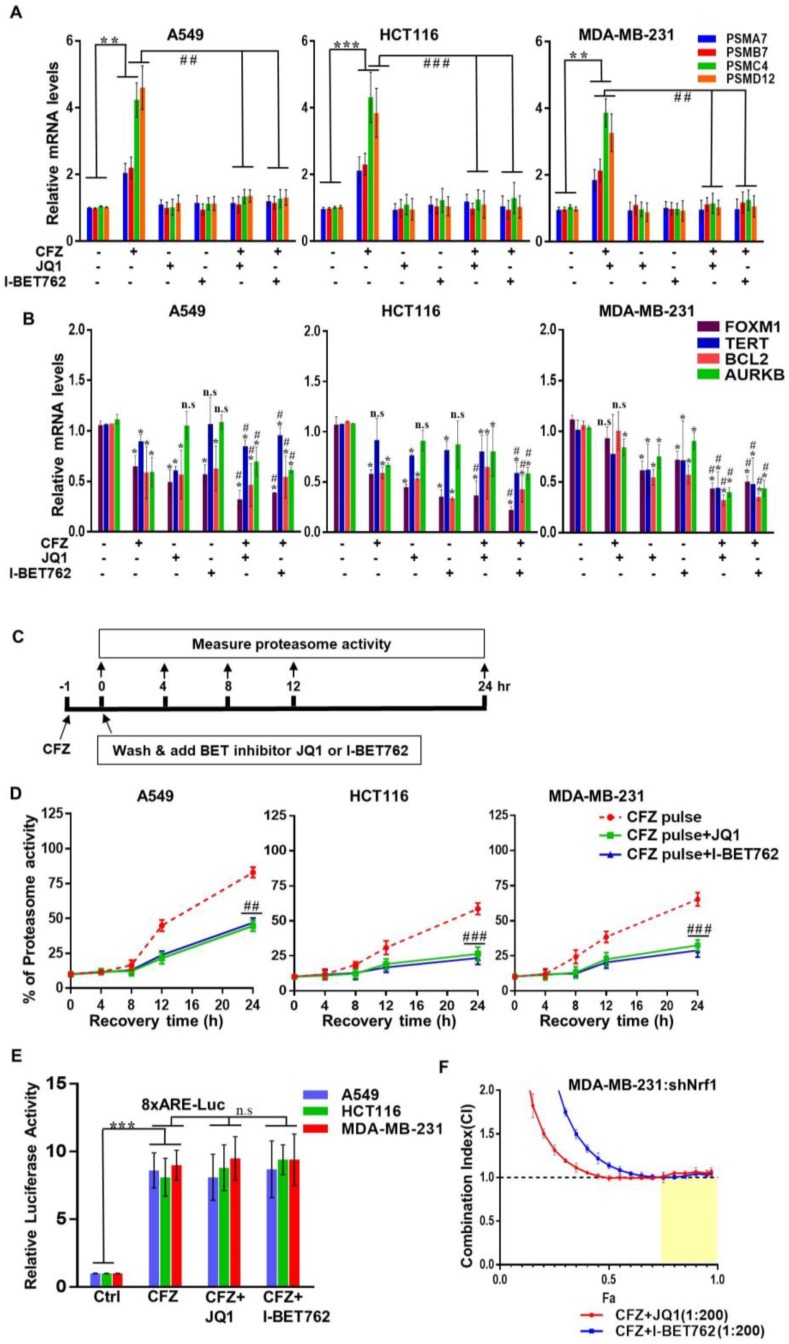
BET inhibitors impair Nrf1-mediated induction of proteasome genes and recovery of proteasome activity in response to CFZ. The A549, HCT116, and MDA-MB-231 cells were treated with CFZ (200 nM) alone and in combination with I-BET762 (10 µM) and JQ1 (1 µM) for 8 h. The DMSO treatment was used as vehicle control. Following treatments, RNA was extracted from cells and analyzed for representative proteasome subunit (**A**), BET target (**B**) mRNA levels with gene specific primers using quantitative RT-PCR. The 18s rRNA tanscript levels were used for normalization. Error bars denote SD (*n* = 3); (**C**) A schematic representation of the proteasome recovery assays is shown; (**D**) A549, HCT116, and MDA-MB-231 cells were treated with 20 nM CFZ for 1 h (pulse treatment). The drugs were, then, washed out and the cells were allowed to recover in the absence or presence of I-BET762(10 µM) and JQ1 (1 µM) for 0, 4, 8, 12, and 24 h. Proteasome activity was measured at indicated time points and normalized to DMSO treated control cells. Error bars denote SD (*n* = 3); (**E**) A549, HCT116, and MDA-MB-231 cells were transiently transfected with a firefly luciferase construct driven by eight repeats of the antioxidant response element (8xARE-Luc) along with a renilla luciferase construct. Forty-eight hours after transfection, cells were treated overnight with CFZ (200 nM) alone or in combination with JQ1 (1 µM) or I-BET762 (10 µM) as indicated. Dual luciferase assays were, then, performed to measure the firefly and renilla luciferase activity values. Normalized luciferase activity is shown. Error bars denote SD (*n* = 3); (**F**) MDA-MB-231-shNrf1 cells were treated with different doses of CFZ (0.5, 2, 8, and 32 nM), along with one of the BET inhibitors (I-BET762, JQ1) in different doses (0.1, 0.4, 1.6, and 6.4 μM) for 72 h. The combination index (CI) and fraction affected (Fa) values were determined using CompuSyn software from cell viability data, and are shown in the graph. The results are shown as mean ± SD, n = 3. CI < 1.0 indicates synergism, CI = 1.0 indicates additive effect, and CI > 1.0 indicates antagonism. *, *p* < 0.05, **, *p* < 0.005, ***, and *p* < 0.0005 as compared with controls; #, *p* < 0.05, ##, *p* < 0.005, and ###, *p* < 0.0005 as compared with the CFZ-treated group.

To explore possible mechanisms behind the synergy of proteasome and BET inhibitors, first, we sought to examine the Nrf1 pathway. We and others have previously established Nrf1 as a master transcription factor of the proteasome genes [12,14,40]. In response to proteasome inhibition, Nrf1 is activated resulting in de novo synthesis of proteasome genes leading to a bounce-back response or recovery of proteasome activity [12]. Here, using three different cancer cell lines, we investigated the changes in proteasome gene transcription in response to CFZ and BET inhibitors JQ1, and I-BET762. We found that in all these cell lines, CFZ treatment alone resulted in a robust induction of representative proteasome genes as compared with the control (Figure 2A). Interestingly, this induction was completely abolished when either JQ1 or I-BET762 was added along with CFZ, suggesting that these BET inhibitors could be antagonizing Nrf1-mediated transcription of its target genes. Of note, treatment with BET inhibitors alone did not elicit any appreciable changes in basal expression of proteasome genes (Figure 2A). Taken together, our data indicate that BET inhibitors block Nrf1-mediated induced expression of proteasome genes.

Next, as controls under our treatment conditions, we examined the mRNA levels of FOXM1, TERT, BCL2, and AURKB which are some of the target genes of BET proteins [41,42]. Interestingly, in contrast to proteasome genes, we observed a modest decrease in BET target genes in response to CFZ alone (Figure 2B). These genes also exhibited a decrease after treatment with BET inhibitor alone, and depending on the cell type, registered a further decline with CFZ + BET inhibitor (Figure 2B). However, this was quite a contrast to the consistent increase in proteasome genes in response to CFZ and the subsequent attenuation of this increase in response to CFZ + BET inhibitor which we observed earlier across all three cell lines examined (Figure 2A), implying that impaired induction of proteasome genes could be a major characteristic of CFZ + BET inhibitor combination.

Next, to understand the impact of BET inhibitors in this context on a functional level, we turned to proteasome recovery assays that measure the ability of the cells to bounce back from proteasome inhibition [12,15,43]. It is important to note that CFZ binds irreversibly to the proteasome β5 active site, whereas BTZ and IXA binding are reversible. We have previously demonstrated that in cells pulse treated with an irreversible proteasome inhibitor, the bounce-back response of proteasome activity almost exclusively relies on the Nrf1 pathway [12]. Taking advantage of these facts, here, we treated three different cancer cells with CFZ for an hour such that the residual proteasome activity was ~10% as compared with the vehicle control. Then, we washed away the excess CFZ and followed the proteasome activity for 24 h, either in the absence or presence of BET inhibitors JQ1 and I-BET762 (Figure 2C). Whereas the cells pulse treated with CFZ regained their proteasome activity steadily in the subsequent 24 h washout period, the extent of recovery was significantly impaired in the cells pulse treated with CFZ followed by JQ1 or I-BET762 exposure (Figure 2D). Overall, our results establish BET inhibitors as potent antagonists of the Nrf1 pathway.

Given that Nrf1 is an endoplasmic reticulum (ER)-bound transcription factor, its activation in response to proteasome inhibition involves a series of steps including ATPase p97/VCP-dependent retrotranslocation into the cytosol [13], followed by the protease DDI2-mediated proteolytic processing [44,45], and the mobilization of the active form to the nucleus where it can bind to proteasome gene promoters to activate their transcription. It is possible that the BET inhibitors could interfere with one or more of these steps to thwart Nrf1 activity. Alternatively, it could be that Brd2/Brd3/Brd4 (BET inhibitor targets) act as necessary cofactor(s) of Nrf1 akin to the TIP60 complex that we recently demonstrated [15]. However, when we utilized a luciferase construct driven by a synthetic promoter harboring eight repeats of the antioxidant response element (ARE, the DNA sequence that Nrf1 binds to), we observed no reduction in the CFZ-induced luciferase activity from cells that were coincubated with JQ1 or I-BET762 (Figure 2E). This suggests that although CFZ-induced Nrf1 remains transcriptionally competent in the presence of BET inhibitors in general, it is unable to transactivate proteasome genes via their natural promoters in this context, the mechanism of which is currently unclear.

Next, we wondered if inactivation of Nrf1 plays a significant role in the synergistic cell death that we observed earlier in the CFZ + BET inhibitor treatments. To this end, we used MDA-MB-231 cells with shRNA-mediated Nrf1 knockdown (shNrf1) and found that treatments with CFZ + JQ1 or CFZ + I-BET762 were not synergistic (Figure 2F), thus, ascribing a critical role for Nrf1 inactivation in this context. 

### 3.3. BET Inhibitors Exacerbate CFZ-Mediated Unfolded Protein Response (UPR)

Inhibition of the proteasome results in the accumulation of misfolded and short-lived proteins which leads to ER stress [46]. A major consequence of the ER stress is the activation of the UPR pathway [47] which is generally categorized into three branches each of which is mediated by a different ER protein, i.e., PERK, IRE1, or ATF6. Although UPR signaling initially acts in a cytoprotective fashion, in the face of sustained ER stress, it can lead to cell death [47]. 

**Figure 3:**
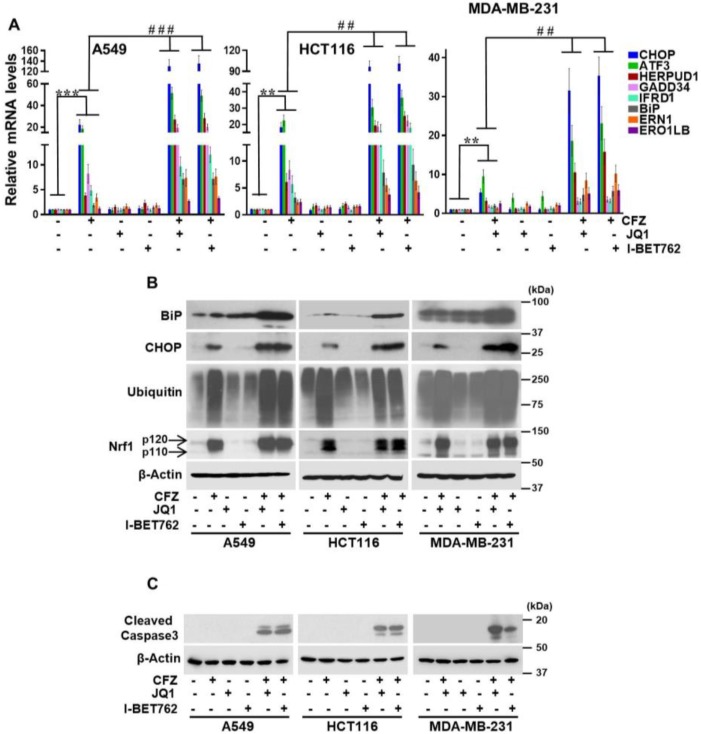
Combination of proteasome and BET inhibitors results in exacerbation of the UPR. (**A**) BET inhibitors (JQ1 1 µM and I-BET762 10 µM) and proteasome inhibitor (CFZ 200 nM) were added either alone or in combination to A549, HCT 116, and MDA-MB-231 cells for 8 h, as indicated. DMSO was used as the vehicle control. RNA was isolated from these cells and subjected to quantitative RT-PCR to measure transcript levels of select stress response genes, as shown. The mRNA levels of 18s rRNA were used for normalization. Error bars denote SD (*n* = 3); (**B**) Cells treated as above were used for Western blot analysis employing specific antibodies as indicated and β-Actin was used as the loading control. The experiments were performed three independent times and a representative blot is shown; (**C**) A549, HCT116, and MDA-MB-231 cells were treated with BET inhibitors (JQ1 1 µM and I-BET762 10 µM) and proteasome inhibitor (CFZ 200 nM), similar as above, for 14 h and lysates were used to analyze cleaved caspase-3 using specific antibody. β-Actin served as the loading control. A representative blot of three independent experiments is shown. *, *p* < 0.05, **, *p* < 0.005, ***, and *p* < 0.0005 as compared with controls; #, *p* < 0.05, ##, *p* < 0.005, ###, and *p* < 0.0005 as compared with the CFZ-treated group.

Here, using different cancer cell lines, we sought to characterize the magnitude of the UPR in response to CFZ and CFZ+BET inhibitors by quantifying the changes in mRNA levels of representative genes in the PERK branch (ATF3, CHOP, and GADD34), IRE1 branch (IFRD1, ERN1, and ERO1LB), and the ATF6 branch (HERPUD1 and BiP). As expected, in these cancer cell lines, we observed an appreciable increase in all of these UPR genes in response to CFZ alone (Figure 3A). Strikingly, when these cells were cotreated with BET inhibitors JQ1 or I-BET762 in addition to CFZ, the increase in the UPR genes was significantly enhanced. Most noteworthy was the level of enhancement in CHOP mRNA in A549 (~20-fold in CFZ vs. ~120-fold in CFZ + BET inhibitor) and HCT116 (~20-fold in CFZ vs. ~90-fold in CFZ + BET inhibitor) cell lines (Figure 3A). In line with these observations, GADD34, a downstream target gene of CHOP, also exhibited a similar trend (Figure 3A).

Overall, the level of enhancement of UPR transcript levels in CFZ + BET inhibitor-treated samples as compared with CFZ-treated samples was maximal in A549, followed by HCT116 and MDA-MB-231, and this matched up with the differing levels of CFZ + BET inhibitor synergism that we observed in these cell lines (Figure 1B). Interestingly, treatment with either BET inhibitor alone did not show significant changes in the UPR genes (with one exception in MDA-MB-231 where ATF3 mRNA increased approximately four-fold in response to JQ1/I-BET763). Together, our results indicate that BET inhibitors have the ability to aggravate the UPR initiated by proteasome inhibition. This notion was further reinforced when we examined CHOP and BiP at the protein level. We saw that the levels of these proteins were significantly enhanced in the cells treated with CFZ + BET inhibitors as compared with CFZ alone (Figure 3B). As a control, we examined the accumulation of ubiquitinated proteins, which increased in response to CFZ and CFZ + BET inhibitors, as expected (Figure 3B). We also monitored the levels of the Nrf1 protein under these conditions and saw that both p120 (inactive precursor) and p110 (proteolytically processed and active) forms accumulated in response to CFZ and this pattern did not change in the CFZ + BET inhibitor treated cells (Figure 3B), implying that BET inhibitors do not interfere with Nrf1 protein levels.

Next, we compared the level of apoptosis in response to treatments with CFZ alone and CFZ + BET inhibitors, and found that in combination treatments, cleaved caspase-3 levels were markedly increased (Figure 3C), consistent with the synergistic cell death that we observed earlier (Figure 1B) under similar conditions.

### 3.4. Depletion of Nrf1 Exacerbates CFZ-Mediated UPR

**Figure 4:**
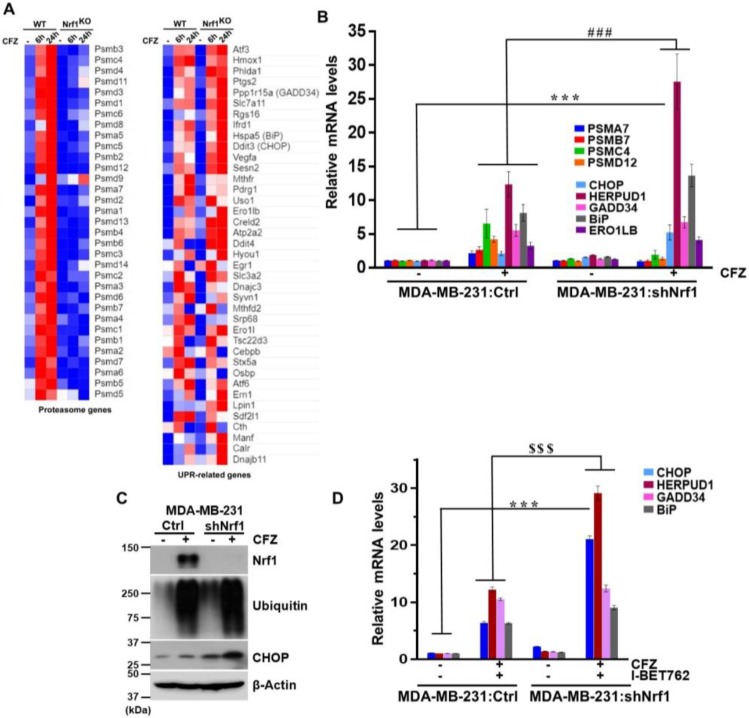
Depletion of Nrf1 leads to exacerbation of UPR under proteotoxic stress conditions. (**A**) Wild-type (WT) and Nrf1 knock-out (KO) NIH-3T3 cells were treated or not with 200 nM CFZ for 6 or 24 h and the samples from 3 biological replicates were analyzed by RNA-seq. Heat maps for changes in proteasome and UPR-related genes are shown and were generated using the Morpheus software (https://software.broadinstitute.org/morpheus/). A relative color scheme is employed where the minimum and maximum values in each row were used to convert values to color. The relative levels are indicated by varying color intensities of blue (low) and red (high); (**B**) MDA-MB-231 cells expressing a vector (Ctrl) or shNrf1 were treated with CFZ (200 nM) for 8 h and RNA was extracted and analyzed for indicated proteasome and UPR-related mRNA levels using quantitative RT-PCR with gene specific primers. The levels of 18s rRNA were used for normalization. Error bars denote SD (*n* = 3); (**C**) From cells treated as above, whole cell lysates were analyzed by immunoblotting for Nrf1, ubiquitin, and CHOP levels. β-Actin was used as loading control; (**D**) **The** MDA-MB-231:Ctrl and MDA-MB-231:shNrf1 cells were cotreated with CFZ (200 nM) and I-BET762 (10 µM) for 8 h and total RNA was isolated and analyzed for mRNA levels of UPR target genes using quantitative RT-PCR. The 18s rRNA levels were used for normalization. Error bars denote SD (*n* = 3). ***, *p* < 0.0005 as compared with the controls; ###, *p* < 0.0005 as compared with the CFZ-treated group. $$$, *p* < 0.0005 as compared with the CFZ + I-BET762 treated group.

Thus far, our results indicate that BET inhibitors attenuate the ability of Nrf1 to induce proteasome genes and, at the same time, also exacerbate the UPR caused by proteasome inhibition. We wondered if these two seemingly disparate results could somehow be related. To address this question, we used a previously characterized NIH-3T3 cell line where Nrf1 has been knocked out (KO) using CRISPR/Cas9 [15,16]. We treated both control wild-type (WT) and Nrf1^KO^ cell lines for either 6 or 24 h with CFZ and analyzed the changes in gene expression using RNA-sequencing. Consistent with an established role for Nrf1 in inducing proteasome genes [12,14], we saw a robust increase in these genes in the WT, but not in the Nrf1^KO^ cells in response to CFZ (Figure 4A). We also observed a general increase in UPR-related genes in the WT cells in response to CFZ. Strikingly, under these conditions in the Nrf1^KO^ cells treated with CFZ, we saw that a number of those UPR-related genes were hyperinduced (Figure 4A).

Next, we sought to verify these results in MDA-MB-231 cells with shRNA-mediated Nrf1 knockdown (shNrf1). In response to CFZ, we saw that the proteasome genes were induced in an Nrf1-dependent fashion, and UPR-related genes were hyperinduced in shNrf1 cells as compared with the vector control cells (Figure 4B). Consistent with these results, we observed that in response to CFZ, the CHOP protein levels in shNrf1 cells were significantly elevated as compared with the vector control cells (Figure 4C). Together, our results point to a model in which loss of Nrf1 aggravates the UPR caused by the inhibition of the proteasome. 

Taking this one step further, we examined some of the UPR-related genes in MDA-MB-231: shNrf1 cells treated with the CFZ + BET inhibitor combination. We found that in response to the CFZ + I-BET762 treatment, whereas GADD34 and BiP were similarly induced in both the vector control and shNrf1 cells, CHOP and HERPUD1 were induced more in the shNrf1 cells (Figure 4D). These results suggest that some, but not all, of the CFZ-responsive UPR-related genes are hyperinduced by BET inhibitors via Nrf1 inactivation.

## 4. Discussion

Proteasome inhibitors have emerged as effective therapeutics in multiple myeloma and mantle cell lymphoma [5]. However, their efficacy in other cancer types, especially solid tumors, remains very limited. Here, we found that BET inhibitors such as JQ1, I-BET762, and I-BET151 have the ability to potentiate the cytotoxicity of proteasome inhibitor drug carfilzomib in multiple solid tumor cell lines. 

BET inhibitors, as the name implies, target and inhibit the BET family of proteins that consists of germ cell-specific BRDT and ubiquitously expressed Brd2, Brd3, and Brd4 [48]. Using their bromodomains, these BET proteins recognize and bind acetylated lysine residues on histones, recruit factors associated with transcription such as P-TEFb, and act as positive regulators of gene transcription in most scenarios. For instance, Brd4, a well-studied member of the BET family, is known to activate genes involved in cell growth and cell cycle progression which are some of the prominent features associated with cancer cells [48]. Thus, there is intense interest in developing BET inhibitors as anticancer therapeutics. Our current study supports the use of BET inhibitors in conjunction with proteasome inhibitors.

Our results show that one of the advantages of this combination is the inactivation of the cytoprotective Nrf1 pathway that otherwise acts to induce proteasome gene expression in response to proteasome inhibition. Intriguingly, whereas the activity of BET proteins seems to be necessary to support Nrf1-mediated proteasome gene transcription after proteasome inhibition, it appears to be dispensable for Nrf1-dependent transcription from a synthetic promoter, suggesting a context-dependent requirement for BET proteins. The precise molecular events that underlie this effect remain to be elucidated.

Apart from the inactivation of the Nrf1 pathway, another potential explanation for the synergy between proteasome and BET inhibitors is the exacerbation of UPR signaling that was evident in the carfilzomib + BET inhibitor treatments as compared with carfilzomib alone. Of all the UPR-related genes that we tested, the level of CHOP was the most noteworthy and was significantly hyperinduced in the carfilzomib + BET inhibitor treatments as compared with carfilzomib alone. Given that CHOP is a well-established pro-apoptotic protein [49], it could very well be a key driver of the synergistic cell death that we observed in this context.

Proteasome inhibitors are well known for inducing UPR [6]. Our results show that although BET inhibitors by themselves do not cause UPR, they seem to aggravate the response initiated by proteasome inhibition. Intriguingly, our study also shows that the UPR is hyperactivated in Nrf1-deficient cells. Taken together, our data suggest that the ability of BET inhibitors to attenuate Nrf1-mediated proteasome bounce-back response could in part explain their ability to exacerbate the UPR triggered by proteasome inhibition. Overall, our study offers a mechanistic explanation for the synergy between proteasome and BET inhibitors and provides a rationale for exploring this combination further via in vivo xenograft studies and possibly in future clinical trials. 

## Data Availability

The RNA-sequencing data discussed in this publication have been deposited in the NCBI’s Gene Expression Omnibus (GEO) and are available through accession number GSE144817.

## Supplementary Materials

The following are available online at https://www.mdpi.com/2218-273X/10/4/501/s1, Table S1: Primers used in quantitative reverse transcription PCR.
