# Supplementary material for: BET Inhibitors Synergize with Carfilzomib to Induce Cell Death in Cancer Cells via Impairing Nrf1 Transcriptional Activity and Exacerbating the Unfolded Protein Response
Source: Biomolecules. 2020 Mar 26;10(4):501. doi: 10.3390/biom10040501 (PMC7226130; doi:10.3390/biom10040501)
Supplement: Supplementary file 1 [file biomolecules-10-00501-s001.pdf]

biomolecules
MDPI

Supporting Information

Table S1: Primers used in quantitative reverse transcription PCR

Human Gene	Forward Primer (5’-3’)	Reverse Primer (5’-3’)
GADD34	ACCCTTTCTTCCTCCTGTCC	CCACTGTCTTCAGCCTCCTC
CHOP	CTTTCTCCTTCGGGACACTG	TGTGACCTCTGCTGGTTCTG
HERPUD1	CTGGGAAGCTGTTGTTGGAT	TCAGGATACTGTCCCCGATT
BiP	TCCTATGTCGCCTTCACTCC	ACGGCAAGAACTTGATGTCC
ATF3	GTGCCGAAACAAGAAGAAGG	TGGAGTCCTCCCATTCTGAG
ERO1LB	TCCGGTTGGAATAAAAGCTG	CACAAAAGTGATCCCGTGAA
IFRD1	GAAGACAAGGCAAGCAGCTC	GGCCCAGCTGAATACAAAGA
ERN1	CACAGTGACGCTTCCTGAAA	AGCGTATACAGGCTGCCATC
PSMA7	CTGTGCTTTGGATGACAACG	CGATGTAGCGGGTGATGTACT
PSMB7	TGCAAAGAGGGGATACAAGC	GCAACAACCATCCCTTCAGT
PSMC4	GGAAGACCATGTTGGCAAAG	AAGATGATGGCAGGTGCATT
PSMD12	GTGCGCGACTGACTAAAACA	TAGGCAGAGCCTCATTTGCT
18S rRNA	ATGGCCGTTCTTAGTTGGTG	CGCTGAGCCAGTCAGTGTAG
FOXM1	TTCACAGCATCATCACAGCA	CCTAGCTGCAGGTTTTGGTC
TERT	GAAGAACGTGCTGGCCTTC	GAGCCACCAGCACAAAGAG
BCL2	GAGGATTGTGGCCTTCTTTG	ACAGTTCCACAAAGGCATCC
AURKB	GTGTACTTGGCTCGGGAGAA	CAGCTCTTCTGCAGCTCCTT
